# Secondary ocular hypertension due to tentorial dural arteriovenous fistula: a case report

**DOI:** 10.1186/s12886-023-03124-8

**Published:** 2023-09-11

**Authors:** Yao Ma, Kun Lv, Kangyi Yang, Huijuan Wu

**Affiliations:** 1 Department of Ophthalmology, Peking University People’s Hospital, No.11 Xizhimen South Street, Xicheng District, 100044 Beijing, China; 2 Eye diseases and optometry Institute, Beijing, China; 3 Beijing Key Laboratory of Diagnosis and Therapy of Retinal and Choroid Diseases, Beijing, China; 4 College of Optometry, Peking University Health Science Center, Beijing, China

**Keywords:** Tentorial dural arteriovenous fistulas, Ocular hypertension, Proptosis, Gamma knife

## Abstract

**Background:**

Tentorial dural arteriovenous fistulas (TDAVFs) are abnormal shunts between meningeal arteries and the intradural venous system located in the tentorial dura mater, which typically manifest with haemorrhage or progressive neurological disorders. TDAVFs with pure ocular presentation have been rarely reported.

**Case Presentations:**

The case of a 56-year-old man presented with unilateral eye redness, proptosis and elevated intraocular pressure was reported herein, which was caused by a TDAVF. The fistula was fed by the left posterior cerebral artery and posterior meningeal artery. The drainage was into the basal vein and internal cerebral veins, which led the arterial blood flow forward to the left superior ophthalmic vein directly. The redundant blood flow caused the rise of episcleral venous pressure, leading to the clinical presentations. Gamma knife radiosurgery was performed then considering the delicate vascular structure and its deep location. The corkscrew hyperaemia was gradually alleviated after the surgery, but the intraocular pressure remained elevated at follow-ups.

**Conclusion:**

Dural arteriovenous fistulas which are not directly connected to cavernous sinus could cause ocular presentations like proptosis, eye redness and ocular hypertension.

**Supplementary Information:**

The online version contains supplementary material available at 10.1186/s12886-023-03124-8.

## Background

Secondary ocular hypertension is a group of disorders in which the rise of intraocular pressure (IOP) is associated with the primary ocular or systemic diseases. Ocular hypertension due to elevated episcleral venous pressure (EVP) is relatively rare [1]. Patients with dural arteriovenous fistulas (DAVFs) primarily experience neurological symptoms [2]. Ocular manifestations are uncommon and mostly associated with DAVFs in cavernous sinus. Tentorial dural arteriovenous fistulas (TDAVFs) are DAVFs located in the tentorial dura mater, typically accompanied by intracerebral haemorrhage or progressive neurological deficit (97%) [3]. To our knowledge, there have been reported few cases of TDAVFs primarily presenting with unilateral proptosis and ocular hypertension.

## Case presentations

A 56 years old male presented to our ophthalmology department with complaints of 9-month redness and proptosis in the left eye. No apparent visual impairment, distorted vision, or neurological symptoms were reported. The patient had no history of head or ocular trauma and had been diagnosed with Hashimoto’s thyroiditis for 5 years.

**Fig. 1:**
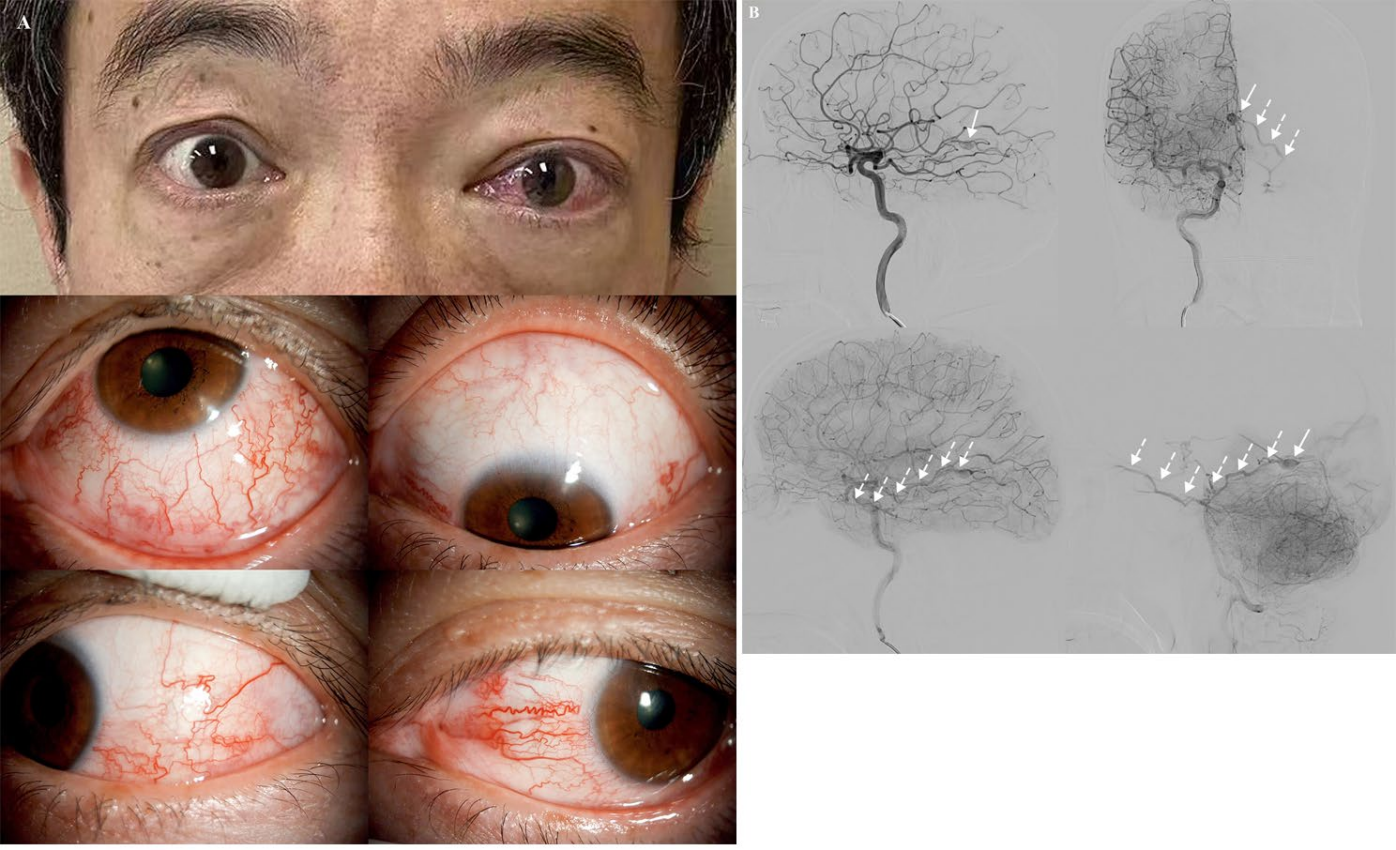
**A** Proptosis and corkscrew hyperaemia with dilated scleral vessels were observed in the left eye. **B** DSA indicated a TDAVF (*white arrows*) fed by the left posterior cerebral artery and posterior meningeal artery, draining directly to the left superior ophthalmic vein without direct connections with the cavernous sinus (*dotted arrows*)

On examinations, visual acuity was 20/20 for both eyes, and the IOP was 12mmHg and 25mmHg in the right and left respectively. Non proptosis was noted in the left eye without lid retraction or eye movement restrictions. The exophthalmometer readings were 14 mm and 19mm for the right and left eye. Corkscrew hyperaemia with dilated scleral vessels was observed in the left eye (*Figure *1A). Gonioscopy showed open angles in both anterior chambers and no obvious lesions were found in the optic disc or fundus vessels bilaterally. Visual fields examination showed no visual field loss and optical coherence tomography (OCT) indicated no optic nerve rim loss or notch, and no retinal nerve fiber layer defects. Normal extraocular muscles were shown by ultrasonography. Magnetic resonance imaging (MRI) of head and orbit indicated no space-occupying orbital abnormality.

Suspecting a secondary ocular hypertension caused by abnormal vascular malformations (AVMs), cranial magnetic resonance angiography (MRA) and diagnostic digital subtraction angiography (DSA) were further performed. MRA indicated a right embryonic posterior cerebral artery and an open left posterior communicating artery. DSA showed a TDAVF fed by the left posterior cerebral artery and posterior meningeal artery (PMA). The drainage of the malformed vascular structure was into the basal vein and internal cerebral veins, leading the arterial blood flow forward to the left superior ophthalmic vein (*Figure* 1*B*, *video*).

Endovascular surgery was considered of high risk due to the delicate nature of the vascular malformation and its deep location. Therefore, gamma knife radiosurgery (GKR) was performed with a dose of 32.7Gy at the center and 18Gy at the periphery. Carteolol hydrochloride and brimonidine tartrate eye drops were also prescribed for the left eye to lower IOP. The corkscrew hyperaemia was gradually alleviated after the surgery, but the IOP remained elevated. At the six-month follow-up, IOP in the left eye was 24mmHg, but there was still no evidence of visual field loss or retinal nerve fiber layer impairment.

## Discussion and conclusions

The elevated IOP with an open angle could have been mistaken for primary open-angle glaucoma, but the corkscrew hyperaemia with dilated scleral vessels suggested the need to rule out intracranial AVMs. In addition, the history of Hashimoto’s thyroiditis also raised the possibility of thyroid-associated ophthalmopathy (TAO). According to previous study, TAO is generally associated with Graves’ disease and rarely with Hashimoto’s thyroiditis [4]. In approximately 40% of patients with TAO, systemic and ocular presentations have a simultaneous onset. The most common clinical sign is lid retraction (90%), followed by proptosis (60%) and eye movement restrictions (40%). However, it should be noted that researchers have postulated the enlargement of superior rectus muscle alone may cause reduced venous outflow through simple external compression and/or periphlebitis of the superior ophthalmic vein [5]. In the present case, the patient’s asymmetric proptosis might have been a source of confusion. However, no significant imaging findings indicated orbital congestion, increased fat volume, or enlarged extraocular muscles.

Common causes of ocular hypertension related to elevated EVP can be listed as followings: AVMs, venous obstruction (including retrobulbar tumor and thyroid-associated orbitopathy), superior vena cava syndrome and idiopathic dilated episcleral vessels. Among AVMs, DAVFs in cavernous sinus and carotid cavernous fistulas (CCF) were closely related to ocular manifestations [6]. Other types of AVMs with ophthalmic presentation have also been reported, such as DAVFs supplied by ascending pharyngeal artery, sphenoparietal DAVF, [7] and AVMs in the right parietal-occipital area [8]. DSA remains the gold standard for evaluating and detecting the detail of AVMs. In these cases, retrograde arteriovenous mixed blood eventually flows into superior ophthalmic vein through different indirect aberrant drainage pathways, resulting in unilateral eye redness, proptosis, recurrent headache, etc. In this case, the shunt was located in the tentorium cerebelli area without direct connections with the cavernous sinus. As shown by DSA, the abnormal vascular structure was fed by the left posterior cerebral artery and PMA (arising from the left vertebral artery), draining into the basal vein and internal cerebral veins. Then the left superior ophthalmic vein collected the redundant blood flow, leading to an increase in EVP and resulting in clinical presentations such as elevated IOP, proptosis and tortuously dilated scleral vessels.

Previous studies have shown that TDAVFs are mainly supplied by branches of the meningohypophyseal trunk, middle meningeal artery (MMA), PMA and occipital artery [9]. Besides, they frequently have angiographic features associated with hemorrhage, which could lead to severe neurological sequelae [10]. Ocular presentations of TDAVFs are uncommon, as they typically occur simultaneously with neurological symptoms and signs. Liang-Fu Zhou et al. recorded two TDAVFs with diplopia, headache, vertigo and ataxia. Goetz’s group also reported an unusual TDAVF case that a 38-year-old female with long-standing bilateral proptosis experienced sudden headache and visual disturbances [11]. Angiography revealed a DAVF supplied by a falx branch from the left vertebral artery and bilateral middle meningeal arteries, which drained directly into the dilated Galen vein through the cavernous sinus and the basal vein into bilateral superior ophthalmic veins. With a different drainage pattern from the case reported here, a 59-year-old man presented with unilateral progressive chemosis and exophthalmos reported by Naotsugu Toki turned out to be a TDAVF that was fed by the MMA and the meningohypophyseal artery, drained into the superior ophthalmic veins and the cerebellar cortical veins via an enlarged petrosal vein [12]. To our knowledge, there have been few reported cases of TDAVFs primarily presenting with unilateral proptosis and ocular hypertension.

Generally, endovascular approach is the first-line treatment for most DAVFs. Stereotactic radiosurgery and surgery remain alternative or even last salvage options, particularly when an endovascular treatment fails or is considered dangerous [13]. In this case, endovascular treatment may be of danger due to the delicate structure and surgically risky location, therefore radiosurgery could be a better option with high obliteration and low mortality rates [14]. The mechanisms of AVMs obliteration after radiosurgery involve progressive intimal thickening, thrombosis of irradiated vessels, and ultimately occlusion of the vascular lumen [15]. Pan et al. have reported a complete obliteration rate of 58% for TDAVFs treated with either radiosurgery exclusively or radiosurgery after failed surgery/embolization [16]. Complete occlusion can usually be obtained 2–5 years after treatment. Further follow-up is still needed to assess the outcome of the surgery.

In conclusion, DAVFs without direct connection to cavernous sinus can cause glaucomatous presentations. In clinical practice, ophthalmologists should take a consideration of intracranial disease when ophthalmic symptoms occur purely and targeted examinations are necessary for diagnosing this type of disease.

### Electronic supplementary material

Below is the link to the electronic supplementary material.

Supplementary Material 1

## Data Availability

Not applicable.

## Abbreviations

AVM: Abnormal vascular malformations
CCF: Carotid cavernous fistulas
DAVF: Dural arteriovenous fistula
DSA: Digital subtraction angiography
EVP: Episcleral venous pressure
GKR: Gamma knife radiosurgery
IOP: Intraocular pressure
TDAVF: Tentorial dural arteriovenous fistula
MMA: Middle meningeal artery
MRA: Cranial magnetic resonance angiography
PMA: Posterior meningeal artery
TAO: Thyroid-associated ophthalmopathy
